# Low-Density Lipoprotein Receptor-Related Protein-1 Signaling in Angiogenesis

**DOI:** 10.3389/fcvm.2017.00034

**Published:** 2017-05-22

**Authors:** Hua Mao, Liang Xie, Xinchun Pi

**Affiliations:** ^1^Department of Medicine, Cardiovascular Research Institute, Baylor College of Medicine, Houston, TX, USA

**Keywords:** angiogenesis, low-density lipoprotein receptor-related protein-1, endothelial cells, proliferation, migration, permeability

## Abstract

Low-density lipoprotein receptor-related protein-1 (LRP1) plays multifunctional roles in lipid homeostasis, signaling transduction, and endocytosis. It has been recognized as an endocytic receptor for many ligands and is involved in the signaling pathways of many growth factors or cytokines. Dysregulation of LRP1-dependent signaling events contributes to the development of pathophysiologic processes such as Alzheimer’s disease, atherosclerosis, inflammation, and coagulation. Interestingly, recent studies have linked LRP1 with endothelial function and angiogenesis, which has been underappreciated for a long time. During zebrafish embryonic development, LRP1 is required for the formation of vascular network, especially for the venous development. LRP1 depletion in the mouse embryo proper leads to angiogenic defects and disruption of endothelial integrity. Moreover, in a mouse oxygen-induced retinopathy model, specific depletion of LRP1 in endothelial cells results in abnormal development of neovessels. These loss-of-function studies suggest that LRP1 plays a pivotal role in angiogenesis. The review addresses the recent advances in the roles of LRP1-dependent signaling during angiogenesis.

## Introduction

Low-density lipoprotein (LDL) receptor-related protein-1 (LRP1, also called α_2_MR or CD91), a member of the LDL receptor family, is involved in not only lipid metabolism but also various pathophysiologic processes such as Alzheimer’s disease, atherosclerosis, inflammation, and coagulation (1). However, the role of LRP1 in endothelial function and angiogenesis remains largely unknown. One reason is that the protein level of LRP1 is low in endothelial cells (ECs) of various vessel beds. In addition, the global LRP1 knockout mouse is embryonic lethal due to embryo implantation defects (2). Endothelial function was therefore underappreciated until inducible knockout models became available. Recent studies have demonstrated that LRP1 expression is dynamically regulated in ECs (3–9). For example, LRP1 can be induced by hypoxia and statins in microvascular ECs. With the help of LRP1 loss-of-function animal models, such as zebrafish with morpholino knockdown and tissue-specific knockout mice, we and other groups demonstrate an emerging role of LRP1 in EC function and angiogenesis, which has been underappreciated for a long time. In this mini-review, we will focus mainly on the recent progresses about its new roles in angiogenesis, especially the regulation of LRP1-dependent signaling pathways in different endothelial processes such as proliferation, migration, and permeability and tube formation.

## LRP1 Domain Structure and Its General Roles in Endocytosis and Cell Signaling

**Figure 1:**
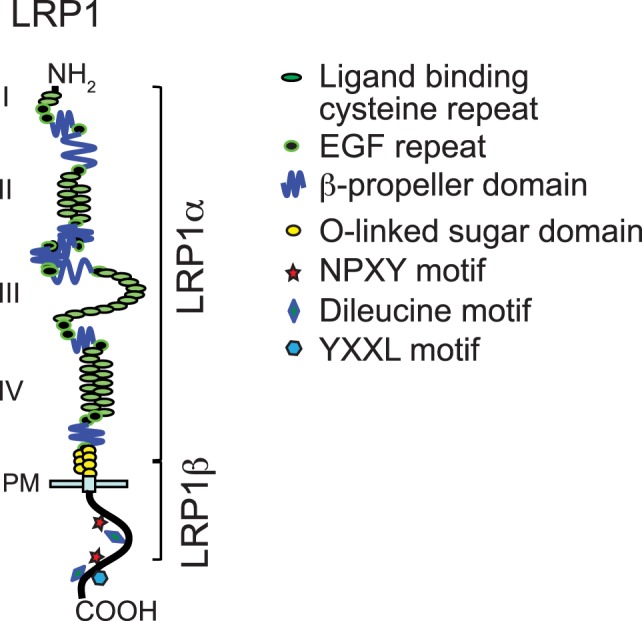
**Modular domain structure of low-density lipoprotein receptor-related protein-1 (LRP1)**. This figure is modified from Figure 1 of a review written by Herz et al. (10).

Low-density lipoprotein receptor family members share similar domain organization patterns, including extracellular ligand-binding domains, a single-pass transmembrane domain, and a cytoplasmic domain (Figure 1) (10). A chaperone called receptor-associated protein (RAP) binds tightly to LRP1 and other LDL receptor family members in the endoplasmic reticulum and blocks the interaction of premature LRP1 and its ligands. The LRP1 precursor (~600 kDa) is cleaved by furin in the trans-Golgi to generate a 515-kDa α chain (LRP1α) possessing four extracellular ligand-binding domains and an 85-kDa membrane-anchored cytoplasmic β chain (LRP1β) that remain non-covalently associated (1, 11, 12). To date, LRP1 binds >40 distinct ligands that are involved in various cellular processes. These ligands are categorized into different groups, including lipoproteins and lipoprotein lipases, proteases and their complex with inhibitors, matrix proteins, and intracellular proteins such as RAP, growth factors, and others. Acting as an endocytic receptor, LRP1 promotes the internalization of associated extracellular ligands through endocytosis and delivers them to the endosomal/lysosomal compartments. After dissociation from ligands, LRP1 is recycled back to the cell surface by interacting with sorting nexin 17 (13). YXXL, dileucine, and NPXY motifs in the cytoplasmic tail of LRP1 serve as dominant endocytosis signals for its highly efficient constitutive endocytosis and recycling (14).

**Figure 2:**
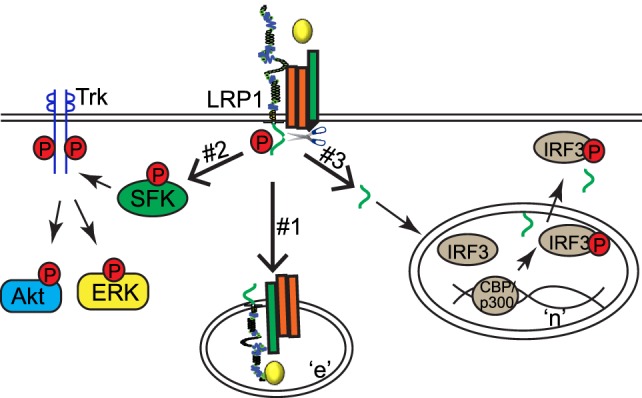
**Low-density lipoprotein receptor-related protein-1 (LRP1) regulates signaling pathways *via* three different mechanisms**. “n,” nucleus; “e,” endosome.

Three different molecular mechanisms have been proposed to explain how LRP1 regulates signaling pathways in response to extracellular stimuli (Figure 2). First, LRP1 acts as an endocytic receptor for signaling complexes (Mechanism #1 in Figure 2). For example, plasma membrane proteins amyloid precursor protein and urokinase receptor (uPAR) undergo endocytosis with the assistance of LRP1 (15, 16). LRP1 also couples with PDGF receptor into endosomes where PDGF receptor can be phosphorylated in response to PDGF (17). Second, some ligands [including tissue-type plasminogen activator (tPA), α_2_-macroglobulin (α_2_M), apoE, and matrix metalloproteinase 9 (MMP9)] may activate cytoplasmic pathways in an LRP1-dependent manner (18–22). For example, the association of ligands tPA or α_2_M with LRP1 leads to the activation of Src family kinase, transactivation of Trk receptors, and activation of cytoplasmic kinases Akt and ERK in neuronal cells (Mechanism #2 in Figure 2) (22). Finally, LRP1 β chain can be further processed by γ-secretase to generate a small fragment-LRP1 C-terminal intracellular domain (LRP1-ICD). LRP1-ICD can be translocated from the cytoplasm to nucleus where it regulates nuclear signaling events. LRP1β processing is dependent on the presenillin-dependent γ-secretase activity (5, 23, 24). For example, the LPS-induced intramembrane proteolysis of LRP1 enables the translocation of its intracellular domain into the nucleus. The nuclear LRP1-ICD protein promotes nuclear export of inflammatory transcription factor interferon regulated factor 3 (IRF3) and therefore negatively regulates transcriptional events of inflammatory genes (Mechanism #3 in Figure 2) (24).

## LRP1 and Angiogenesis

The process of vessel formation occurs as an orderly and tightly regulated event, categorized into vasculogenesis and angiogenesis (25). In contrast to vasculogenesis, the formation of the primitive vessel network from angioblasts, angiogenesis is a remodeling process of an established capillary network, often by sprouting of ECs from preexisting vasculature to generate new capillaries. Angiogenesis happens through five major steps: selective degradation of the basement membrane and surrounding extracellular matrix, EC migration and proliferation, the formation of vascular tubes, and finally the remodeling of the formed vascular network. During the tightly regulated angiogenic process, a delicate balance between pro- [i.e., VEGF, angiopoietin, FGF, bone morphogenetic protein (BMP), sphingosine-1-phosphate (S1P), and urokinase-type plasminogen activator (uPA)] and anti-angiogenic (i.e., angiostatin and endostatin) signaling results in cellular events required for new vessel formation. Dysregulated angiogenesis leads to retinopathy, malignant tumors, and other pathological conditions.

Over the recent years, an increased number of reports demonstrate that LRP1 is expressed in ECs of microvessels and capillaries and involved in endothelial function such as blood–brain barrier transcytosis, permeability, and angiogenesis (3, 5–7, 26–28). LRP1 expression is mainly localized to regions that are active of vasculogenesis in zebrafish (7). LRP1 knockdown in zebrafish results in defects in ventral sprouting events and the formation of caudal vein network. During mouse development, LRP1 mRNA signal distributes ubiquitously during E9.5–12.5 (6). Its protein is detected in the developing brain, heart, and liver that are highly vascularized. When LRP1 is deleted in mouse embryos, vascular developmental defects including defective vasculature with an interrupted endothelial layer and extensive hemorrhage are detected. In a mouse model of oxygen-induced retinopathy, LRP1 depletion in ECs results in increased retinal neovascularization (5). Retinas lacking endothelial LRP1 display increased endothelial proliferation and angiogenic sprouts. In addition, LRP1 regulates cancer cell migration and invasion by upregulating MMP2 and MMP9 expression, AKT and EphA2 activation, and lamellipodia formation (29–31). In the following sections, we will discuss about LRP1-dependent signaling pathways involved in angiogenesis (Table 1).

**Table 1:** **A summary of low-density lipoprotein receptor-related protein-1 (LRP1)-regulated angiogenic pathways**.

Pathway	Study system	Angiogenic phenotype	Reference
Bone morphogenetic protein	Morpholino knockdown in zebrafish	Fewer ventral sprouting events, dorsal and intersegmental vessel malformation, fewer vascular branches	(7)
PARP-1	Tie2-Cre in mice	Increased retinal neovascularization, increased endothelial proliferation	(5)
Sphingosine-1-phosphate	Meox2-Cre in mice	Interrupted endothelial integrity, embryos with extensive hemorrhage	(6)
Urokinase-type plasminogen activator (uPA)	LRP1 Ab and antagonist-receptor-associated protein (RAP) in PMVECs	Inhibition of uPA-induced vascular permeability	(32)
VEGF	Antagonist RAP in HUVEC	Inhibition of VEGF-induced endothelial migration, proliferation, and permeability	(33)

## LRP1 and BMP Signaling

Bone morphogenetic proteins, members of the TGFβ superfamily of proteins, play important roles in many cellular processes such as proliferation, differentiation, motility, and adhesion (34–36). Null mutations of the BMP receptors, BMPs, and its downstream mediators Smads lead to vascular development defects in various animal models (37–44), suggesting a critical role of BMP signaling in angiogenesis. BMP signaling pathway is tightly regulated by extracellular modulators such as BMP-binding endothelial precursor-derived regulator (BMPER). BMPER plays a pivotal role in the development of the embryonic vasculature (45) and tumor invasiveness (46). In ECs, the stoichiometric ratio of BMPER to BMP4 is a key determinant of whether BMP4 signaling is activated or inhibited by BMPER (47). Recent studies demonstrate that LRP1 is a binding partner of BMPER and necessary for optimal BMPER internalization and BMP4 signaling (7). LRP1 also serves as a coreceptor for BMP by interacting with BMP type I receptor ALK6 (7). The specific composition of BMPER/BMP4 receptor complex, including BMPER, BMP4, ALK6, BMPRII, and LRP1, is dynamically regulated by the stoichiometric ratio of BMP and BMPER and the availability of LRP1. LRP1-dependent endocytosis of BMPER and BMP signaling complexes is required for both pro-BMP and anti-BMP activity of BMPER. Many regulators of Bmp signaling have a spatial gradient effect that covers the distance of many cells. The LRP1-dependent endocytosis of BMPER/BMP/BMPR signaling complex (Mechanism #1 in Figure 2) provides a molecular mechanism that operates at the single-cell level.

Low-density lipoprotein receptor-related protein-1 knockdown in zebrafish results in an abnormal vascular phenotype, displaying dorsal and intersegmental vessel malformation, fewer vascular branches within the caudal vein plexus, and a large swollen vascular lumen (7). Disrupted blood flow and slower or stopped heart beat, which are likely secondary due to vascular defects, are also observed in LRP1 knockdown fish. Interestingly, LRP1-deficient fish exhibits fewer ventral sprouting events during venous development. Moreover, the decrease of Smad1/5/8 phosphorylation in LRP1 knockdown fish embryo indicates that BMP signaling is inhibited by LRP1 depletion. These studies suggest that LRP1 regulates angiogenesis through fine-tuning BMP signaling. Additional studies, particularly about endocytic process, should help to characterize how LRP1 promotes the endocytosis of BMP signaling complexes through different routes, either degradative or recycling.

## LRP1 and PARP-1

Poly(ADP-ribose) polymerases (PARPs) play a pivotal role in DNA repair, apoptosis, chromatin modulation, and cell cycle regulation. They catalyze the transfer of ADP-ribose units from NAD+ to acceptor proteins or on PARP itself (48). PARP inhibitors have been developed for cancer treatment. Recent studies indicate that PARP inhibitors may also decrease angiogenesis, either by inhibiting VEGF production or VEGF and basic FGF-induced cellular proliferation, migration, and tube formation (49–51). Recent studies demonstrate that LRP1 negatively regulates new vessel formation through its dynamic interaction with PARP-1 during the process of mouse retinal neovascularization (5). This regulatory mechanism indicates that LRP1 can modulate endothelial processes through the nuclear activity of its intracellular domain (LRP1-ICD; Mechanism #3 in Figure 2). At basal condition, LRP1-ICD has already been processed through regulated intramembrane proteolysis of LRP1 and thereby located in the nucleus. PARP-1 is associated with LRP1-ICD, and its activity is inhibited, which leads to cell arrest at the pre-mitotic stage. In response to hypoxia, the PARP-1 and LRP1 protein complex is dissociated, resulting in a relief of LRP1’s inhibitory effect on PARP-1 activity. This in turn increases the phosphorylation of cyclin-dependent kinase 2 and retinoblastoma, and sequentially cell cycle progression and retinal angiogenesis. These studies suggest that LRP1-ICD is an important player in the intracellular translocation of nuclear proteins such as PARP-1 and IRF3. The generation of free LRP-ICD by proteolysis is likely a control point for this regulatory mechanism. It has been recognized that this proteolysis is gamma-secretase dependent. However, exact protease(s) and detailed molecular mechanisms still need to be determined.

PARP-1 promotes angiogenesis through its promoting role in cell cycle progression. During hypoxia-induced angiogenesis, two events happen with PARP-1, one is an increase in PARP-1 enzymatic activity and the other is its translocation out of the nucleus. It is still a puzzle whether the change in PARP-1 activity is linked with its nuclear export. It is also unclear whether PARP-1 dissociation from LRP1-ICD is required for the nuclear export of LRP1-ICD. The last 33 amino acids within LRP1-ICD are responsible for PARP-1 interaction, where a phosphorylation site for PKA and a dileucine motif are located. However, the effect of PKA and dileucine motif on their interaction and nuclear export remains to be determined.

## LRP1 and S1P Signaling

Sphingosine-1-phosphate is generated from sphingosine, the backbone of sphingolipids on cell membrane. It is an important signaling molecule in many cellular processes including cell growth, anti-apoptosis, and cell migration (52–54). The dysregulation of S1P signaling pathway has been linked to many pathological conditions such as cancer, inflammation, and atherosclerosis (55). S1P forms a complex with albumin, lipoprotein in the circulation and is very abundant in plasma (~0.2–1.1 μM) and lymphatic fluids (~0.1 μM) (56–58). A group of G protein-coupled receptors S1PR (initially called endothelial differentiation gene) act as S1P receptors (59). Depending on different types of small G proteins coupling to S1PRs, S1P regulates different endothelial functions including endothelial permeability, inflammation, migration, and angiogenesis (60). S1PR1 majorly couples to G_i_ to activate phosphatidylinositide 3-kinase and endothelial nitric oxide synthase (eNOS) in order to regulate the integrity of adherens junction and dynamic assembly of focal adhesion complexes. On the other hand, S1P2 and S1P3 can couple to multiple G proteins such as G_i_, G_q/11_, and G_12/13_. Although S1P has been detected inside of cells, the exact role of intracellular S1P remains elusive.

Mice with LRP1 depletion in embryo proper display dramatic vascular defects, including interrupted endothelial integrity, a thin and disorganized smooth muscle cell layer, and an embryo with extensive hemorrhage (6). Mechanistically, Nakajima et al. discover that LRP1 depletion in MEFs relieves the inhibitory effect of S1P on PDGF-BB-induced RAC1 activation and cell migration. The overexpression of LRP1-ICD “rescues” this phenotype of LRP1 depletion. S1P likely blocks PDGF-induced RAC1 activity through G_i_ inhibition, which is suggested by the observation that pertussis toxin restores the inhibitory effect of S1P on PDGF-induced cell migration. Moreover, in HUVECs, LRP1 promotes the inhibitory effect of S1P on PDGF-BB-induced G_i_ activation. It indicates that LRP1 plays a key role in the cross talk between S1P and PDGF-BB signaling and, likely, the final outcome of endothelial migration and angiogenesis. However, it remains a puzzle how LRP1 promotes the inhibitory effect of S1P signaling, specifically, the decrease in G_i_-GTP level. One reasonable explanation is that LRP1 might interact with mediators of S1P/G_i_ pathway, which remains to be identified. It is also intriguing to determine whether LRP1-dependent endocytosis is required for this crosstalk of S1P and PDGF-BB signaling pathways.

## LRP1 and uPAR–uPA–Plasminogen Activator Inhibitor 1 (PAI-1) Signaling

The uPA, its cellular receptor (uPAR), and inhibitor PAI-1 play a requisite role in pericellular proteolysisis. Upon activation, uPA initiates a series of signaling events to modulate cell adhesion, migration, and survival. Once PAI-1 forms a complex with uPA and uPAR, LRP1 binds to this complex and leads to a decrease of uPAR steady-state level (61–63). Recent studies have reported that LRP1 is required for uPA-induced endothelial permeability changes. Although the role of endothelial permeability in angiogenesis is not completely understood, a hypothesis has been around for decades that increased permeability may be required for EC migration (64). Many angiogenic factors such as VEGF induce permeability changes in a fast fashion (1–2 h), likely due to the activation of eNOS and NO formation (65), and increased transcytosis mediated by plasma membrane-derived caveolae (66). VEGF also upregulates uPAR and decreases occludin levels, which leads to a later phase (6–24 h) of permeability increase. VEGF-induced permeability increase could be blocked by both anti-uPA and uPAR antibodies. In addition, the treatment of exogenous uPA increases the flux of tracer molecule (HRP), indicating that uPA is sufficient to increase permeability of retinal microvascular ECs. This uPA-mediated permeability increase is likely paracellular and mediated by the redistribution of junction proteins and decline of electrical resistance. Importantly, the inhibition of LRP1 by RAP significantly suppresses uPA–PAI-1-induced permeability in ECs (32). More signaling studies demonstrate that LRP1 acts as a signaling receptor for uPA–PAI-1-induced G_s_ activation, resulting in the upregulation of cAMP level, and subsequently the activation of PKA and eNOS (Mechanism #2 in Figure 2) (67).

Low-density lipoprotein receptor-related protein-1 also forms a multireceptor complex with VEGFR2, β1-integrin, and uPAR on the cell membrane of ECs and targets this multiprotein complex for endocytosis (Mechanism #1 in Figure 2) (33). Upon VEGF treatment, LRP1 mediates the internalization of this protein complex into the cell. The inhibition of LRP1 by its antagonist RAP blocks the internalization of VEGFR2, and subsequently VEGFR2 phosphorylation and its downstream ERK activation. The inhibition of dynamin-dependent endocytosis by dynasore also has similar inhibitory effect on VEGF signaling, suggesting that LRP1-dependent endocytosis is required for VEGF signaling. The LRP1-dependent endocytosis is further confirmed to play an important role in VEGF-induced EC migration and proliferation in HUVECs. Mechanistically, the interaction of uPAR and LRP1 is crucial for the formation of this multireceptor complex and its internalization. These studies were performed solely with cultured primary ECs. Further *in vivo* studies are needed for fully understanding the significant roles of LRP1 in VEGF–VEGFR2–uPAR signaling at pathophysiologic settings. In addition, the role of LRP1 has only been tested with its internalization inhibitor RAP, which also blocks ligand binding of other LDLR family members (68, 69). More specific inhibitory methods such as deletion mutation of LRP1 or knockdown/knockout techniques will be helpful to clarify the accurate role of LRP1 in endothelial permeability change and VEGF-dependent angiogenesis.

## Concluding Remarks

The role of LRP1 in angiogenesis has just emerged in recent years. Given that LRP1 facilitates the endocytosis of many ligand–receptor complexes and is involved in growth factor or other cytokine-dependent signaling in different pathophysiologic conditions, it is not surprising that various signaling pathways are involved in LRP1-regulated EC growth, migration, and angiogenesis. In multiple angiogenic models, different output of LRP1 loss of function is likely a balanced effect of intricate signaling cascades mediated by LRP1 in ECs as well as in response to different microenvironment settings. Besides the aforementioned signaling pathways that are regulated by LRP1, other pathways are likely involved as well. For example, mouse embryonic fibroblasts or neuronal cells with a knockin mutation of LRP1’s NPxY motif that is responsible for β1-integrin interaction display impaired migratory capability (70). Given that β1-integrin plays an important role in angiogenesis by regulates VEGF signaling, focal adhesions assembly/disassembly, and cytoskeleton remodeling processes [reviewed by Avraamides et al. (71)], it is possible that LRP1 regulates angiogenesis through integrin signaling. During vessel sprouting, key processes including the specification of tip, stalk and phalanx ECs, tip cell migration, and stalk cell proliferation are beginning to be understood (72, 73). It will be interesting to determine whether LRP1 also regulates these processes. Endothelial metabolism, especially PFKBP-driven glycolysis, plays a pivotal role in vessel sprouting of tip cells (74). Whether LRP1, a known regulator of lipid metabolism, regulates endothelial metabolic supply for angiogenic sprouting process could become another interesting research topic. Nevertheless, due to the complex nature of LRP1 signaling, careful analysis of molecular biological assays and *in vivo* pathophysiologic experiments is necessary for the dissection of accurate roles for each signaling pathway in different angiogenic models or pathological conditions of vascular growth. These studies will surely provide useful insights about how to design potent and specific LRP1-based therapeutic strategies for angiogenesis-related diseases.

## Author Contributions

All authors listed have made substantial, direct, and intellectual contribution to the work and approved it for publication.

## Conflict of Interest Statement

The authors declare that the research was conducted in the absence of any commercial or financial relationships that could be construed as a potential conflict of interest.
